# Impact of preventive substrate catheter ablation on implantable cardioverter-defibrillator interventions in patients with ischaemic cardiomyopathy and infarct-related coronary chronic total occlusion: The PREVENTIVE VT randomized multicentre trial

**DOI:** 10.1093/europace/euae109

**Published:** 2024-04-24

**Authors:** David Žižek, Miha Mrak, Matevž Jan, Anja Zupan Mežnar, Maja Ivanovski, Tadej Žlahtič, Nina Kajdič, Bor Antolič, Luka Klemen, Rafael Skale, Jurij Avramovič Gregorič, Jernej Štublar, Andrej Pernat, Matjaž Šinkovec

**Affiliations:** Cardiology Department, University Medical Centre Ljubljana, Zaloška 7, 1000 Ljubljana, Slovenia; Faculty of Medicine, University of Ljubljana, Ljubljana, Slovenia; Cardiology Department, University Medical Centre Ljubljana, Zaloška 7, 1000 Ljubljana, Slovenia; Faculty of Medicine, University of Ljubljana, Ljubljana, Slovenia; University Medical Centre Ljubljana, Cardiovascular Surgery Department, Ljubljana, Slovenia; Cardiology Department, University Medical Centre Ljubljana, Zaloška 7, 1000 Ljubljana, Slovenia; Faculty of Medicine, University of Ljubljana, Ljubljana, Slovenia; Cardiology Department, University Medical Centre Ljubljana, Zaloška 7, 1000 Ljubljana, Slovenia; Cardiology Department, University Medical Centre Ljubljana, Zaloška 7, 1000 Ljubljana, Slovenia; Cardiology Department, University Medical Centre Ljubljana, Zaloška 7, 1000 Ljubljana, Slovenia; Cardiology Department, University Medical Centre Ljubljana, Zaloška 7, 1000 Ljubljana, Slovenia; Cardiology Department, University Medical Centre Ljubljana, Zaloška 7, 1000 Ljubljana, Slovenia; Cardiology Division, General Hospital Celje, Celje, Slovenia; Cardiology Division, General Hospital Izola, Izola, Slovenia; Cardiology Department, University Medical Centre Ljubljana, Zaloška 7, 1000 Ljubljana, Slovenia; Cardiology Department, University Medical Centre Ljubljana, Zaloška 7, 1000 Ljubljana, Slovenia; Cardiology Department, University Medical Centre Ljubljana, Zaloška 7, 1000 Ljubljana, Slovenia; Faculty of Medicine, University of Ljubljana, Ljubljana, Slovenia

**Keywords:** Catheter ablation, Ventricular arrhythmia, Implantable cardioverter-defibrillator, Ischaemic cardiomyopathy

## Abstract

**Aims:**

Primary prevention patients with ischaemic cardiomyopathy and chronic total occlusion of an infarct-related coronary artery (CTO) are at a particularly high risk of implantable cardioverter-defibrillator (ICD) therapy occurrence. The trial was designed to evaluate the efficacy of preventive CTO-related substrate ablation strategy in ischaemic cardiomyopathy patients undergoing primary prevention ICD implantation.

**Methods and results:**

The PREVENTIVE VT study was a prospective, multicentre, randomized trial including ischaemic patients with ejection fraction ≤40%, no documented ventricular arrhythmias (VAs), and evidence of scar related to the coronary CTO. Patients were randomly assigned 1:1 to a preventive substrate ablation before ICD implantation or standard therapy with ICD implantation only. The primary outcome was a composite of appropriate ICD therapy or unplanned hospitalization for VAs. Secondary outcomes included the primary outcome’s components, the incidence of appropriate ICD therapies, cardiac hospitalization, electrical storm, and cardiovascular (CV) mortality. Sixty patients were included in the study. During the mean follow-up of 44.7 ± 20.7 months, the primary outcome occurred in 5 (16.7%) patients undergoing preventive substrate ablation and in 13 (43.3%) patients receiving only ICD [hazard ratio (HR): 0.33; 95% confidence interval (CI): 0.12–0.94; *P* = 0.037]. Patients in the preventive ablation group also had fewer appropriate ICD therapies (*P* = 0.039) and the electrical storms (Log-rank: *P* = 0.01). While preventive ablation also reduced cardiac hospitalizations (*P* = 0.006), it had no significant impact on CV mortality (*P* = 0.151).

**Conclusion:**

Preventive ablation of the coronary CTO-related substrate in patients undergoing primary ICD implantation is associated with the reduced risk of appropriate ICD therapy or unplanned hospitalization due to VAs.

## Graphical Abstract

**Graphical Abstract:**
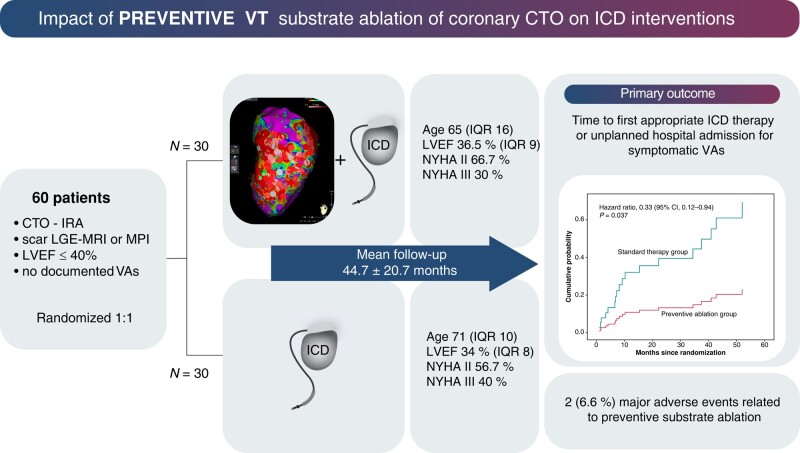
Preventive ablation of coronary total occlusion-related substrate at the time of primary prevention defibrillator implantation is associated with a reduced risk of appropriate defibrillator therapy or unplanned hospitalization due to ventricular arrhythmias.

**What’s new?**

- This is the first randomized trial addressing preventive scar homogenization in a high-risk subset of ischaemic cardiomyopathy patients at the time of primary prevention defibrillator implantation.
- Preventive ablation of the coronary chronic total occlusion-related substrate is associated with the reduced risk of appropriate defibrillator therapy or unplanned hospitalization due to ventricular arrhythmias.
- Patients in the preventive ablation group had fewer defibrillator interventions, arrhythmia-related hospitalizations, electrical storms, and cardiac hospitalizations, while cardiovascular mortality was not significantly reduced.

## Introduction

In patients with ischaemic cardiomyopathy (ICM) implantable cardioverter-defibrillators (ICDs) prevent sudden cardiac death, yet appropriate shocks are associated with reduced quality-of-life and adverse clinical outcomes.^1–4^ Catheter ablation is an established treatment option in the management of patients with ICM and ventricular arrhythmias (VAs).^5–14^ Yet, the optimal timing of the procedure relative to the ICD implantation or documented appropriate therapy is uncertain. Several randomized trials have shown that catheter ablation of the arrhythmogenic substrate at the time of secondary prevention ICD implantation reduces the number of ICD interventions and VA recurrence.^6,10–12^ Recently, the PARTITA trial [Does timing of ventricular tachycardia (VT) ablation affect prognosis in patients with an implantable cardioverter-defibrillator?] showed that catheter ablation after the first ICD shock was associated with not only lower recurrence of VA episodes but also a reduced risk of the composite primary endpoint of total mortality and heart failure (HF) hospitalization.^13^ Therefore, it is conceivable that catheter ablation of the potential arrhythmogenic substrate may be considered in patients eligible for primary prevention ICD, even before experiencing VAs.

Recent studies suggest that patients with chronic total occlusion (CTO) who receive primary and secondary prevention ICDs for ICM have a worse prognosis and are more likely to experience appropriate ICD shocks than those without CTO.^15–17^ Moreover, a subgroup of primary prevention ICD recipients with CTO of an infarct-related artery (IRA-CTO) are at a particularly high risk of appropriate ICD shocks due to fast VTs.^18^ As re-entry mechanism arising from surviving bundles of myocardium within a scar, separated by connective tissue, fibrosis, and disordered intercellular coupling is associated with VT occurrence in most ICM cases,^5^ preventive scar homogenization concept^7^ could present a potential treatment option. Furthermore, revascularization strategies in similar patient populations did not produce a meaningful impact on clinical outcomes.^19,20^

The PREVENTIVE VT trial (Impact of Preventive Substrate Ablation of Coronary Chronic Total Occlusion on Implantable Cardioverter-Defibrillator Interventions) was designed to evaluate whether the preventive scar homogenization reduces the combined endpoint of appropriate ICD therapy or unplanned hospitalization for VAs in high-risk ICM patients with IRA-CTO who did not experience previous VAs and are undergoing primary prevention ICD implantation.

## Methods

### Study design

The PREVENTIVE VT trial was an investigator-initiated randomized controlled trial conducted at four centres in Slovenia between September 2017 and January 2024. While device implantations were performed in all participating centres, catheter ablations were performed in two experienced centres. Details of the participating centres are listed in the Supplementary material online. The Cardiology Department at the University Medical Centre Ljubljana served as the co-ordinating centre for randomization and clinical monitoring. The study was in compliance with the Declaration of Helsinki and approved by the institutional review committees of the participating sites and the Medical Ethics Committee of the Republic of Slovenia (Approval Number: 0120-503/2017/3). The study was supported by an institutional research grant from University Medical Centre Ljubljana. All patients provided written informed consent.

The executive committee designed and conducted the trial which was registered in ClinicalTrials.gov (https://clinicaltrials.gov, NCT03421834). The first author wrote the draft of the manuscript, had full access to all the data in the study, and takes responsibility for its integrity and the data analysis. All the authors made contributions and agreed to submit the manuscript for publication. The data supporting the study findings are available from the corresponding author upon reasonable request.

### Patients

Patients were eligible for inclusion if they had primary prevention indication for ICD with reduced left ventricular ejection fraction (LVEF ≤ 40%) despite optimal medical therapy, had angiographically proven coronary CTO that was associated with previous myocardial infarction (MI), had no previously documented sustained VAs, and were not eligible for revascularization. Detailed inclusion and exclusion criteria are listed in the Supplementary material online.

### Definitions of study variables

Chronic total occlusion (CTO) was defined as total occlusion of the native coronary artery without anterograde flow, with or without anterograde or retrograde filling through collateral vessels.^15–17^ Only CTOs of major epicardial arteries were considered. Infarct-related artery-CTO was defined as the CTO associated with previous MI in the territory of that coronary artery and was preferably confirmed with late gadolinium enhancement at magnetic resonance imaging (MRI). Single-photon emission computed tomography (SPECT) myocardial perfusion imaging could be used if MRI was unavailable. Ventricular arrhythmias included all forms of VTs and ventricular fibrillation. Ventricular tachycardia was defined as a continuous ventricular rhythm with a heart rate above 100 bpm lasting for at least 30 s or requiring termination with ICD device intervention [anti-tachycardia pacing (ATP) or shock] or external defibrillation (in case of arrhythmias with cycle lengths outside the device detection window) or producing symptoms (e.g. syncope). Unplanned VA-related hospitalization was defined as a hospital admission directly related to the VA and occurring within the first 24 h after patient presentation. The arrhythmia needed to be confirmed either by an electrogram stored in the ICD device or an external electrocardiogram strip if it was below the device detection window. Unplanned cardiac hospitalization was defined as hospitalization due to VA or/and worsening HF. An electrical storm was defined as three or more sustained episodes of VAs, each requiring termination, within 24 h.

### Randomization

Eligible patients were randomly assigned 1:1 to a preventive substrate ablation before ICD implantation (preventive ablation group) or ICD implantation only (standard therapy group). Patients and investigators were not blinded to treatment assignment. Randomization was performed with the use of prenumbered, opaque, sealed envelopes in permuted blocks of four and a random number generator. To maintain allocation concealment, the centre-level administrators retained the randomization sequence and investigators were not provided with the assignment until eligibility confirmation. If the patients were assigned to preventive substrate ablation in a centre that did not perform the procedure, they were transferred to the performing centre and then returned for ICD device implantation.

### Catheter ablation procedure

Catheter ablation procedure was always performed before the ICD device implantation. Baseline programmed ventricular stimulation for VA induction before mapping was not mandatory. Myocardial scar related to IRA-CTO and the border zone in the LV were delineated with high-density voltage mapping utilizing a 3-dimensional (3D) electroanatomical mapping system (CARTO^®^ 3, Biosense Webster, Irvine, CA, USA) and a multipolar mapping catheter (PENTARAY^®^, Biosense Webster, Irvine, CA, USA). A minimum of 500 points within the area of interest were required. Any local abnormal EGMs in the form of fractionated (containing four or more sharp deflections), late (two or more sharp deflections separated by an isoelectric segment), and abnormal decrement-evoked local ventricular EGMs unmasked during pacing with ventricular extra-stimuli were tagged on the 3D map and targeted for ablation. Catheter ablation was performed uniformly between different operators and preferably in sinus rhythm with radiofrequency current using an irrigated-tip, contact force sensing ablation catheter (SMARTTOUCH^®^ and SMARTTOUCH^®^ SF, Biosense Webster, Irvine, CA, USA) aiming to abolish abnormal EGMs. No time limit was imposed for mapping and radiofrequency ablation, which remained at the operator’s discretion. Once scar homogenization was completed the elimination of abnormal EGMs was verified with high-density remapping and additional ablations were done when needed. Finally, programmed stimulation with up to three extra-stimuli was performed to test potential VA inducibility. The aim of the ablation procedure was the combined procedural endpoint of abolishing abnormal EGMs and VT non-inducibility with uniform stimulation protocol.

### Device implantation and programming

In the preventive ablation group, the device implantation procedure was carried out no later than 1 week after the ablation procedure, while in the standard therapy group, it was performed no later than 1 week after randomization. Single-chamber ICD devices were recommended unless indicated otherwise (e.g. dual-chamber ICD device or cardiac resynchronization ICD device). The physician had the discretion to select the manufacturer and type of the device. Uniform primary prevention device settings for arrhythmia detection and therapy from different manufacturers were recommended.^21^ Details of device programming are provided in the Supplementary material online.

### Follow-up

Clinical visits were scheduled at 1 and 6 months after ICD implantation and every 6 months thereafter. Patients were followed until the study ended and for at least 24 months. Each visit consisted of a physical examination, device interrogation, history of potential hospital admissions, and evaluation of VA episodes. The use of amiodarone and other antiarrhythmics, except for beta-blockers, was discouraged apart from bridge to ablation after an electrical storm or patient refusal for ablation. In case of electrical storm during follow-up, angiography was always performed.

### Outcomes

The primary outcome was a composite of appropriate ICD therapy or unplanned hospitalization admission for symptomatic VA. Secondary outcomes included each of the primary endpoint components, the incidence of appropriate ICD therapies, unplanned VA-related hospitalization, electrical storm, cardiac hospitalization (due to VA or worsening HF) and cardiovascular (CV) mortality. All periprocedural complications were recorded. Events eligible for primary and secondary outcomes were reported by investigators and adjudicated by the dedicated event committee blinded to treatment assignments.

### Statistical analysis

Based on the previous data on the occurrence of VAs in patients with IRA-CTO,^18^ we projected the primary endpoint to occur in at least 53% of patients randomized to the standard therapy group. Considering the results of the SMASH-VT study^6^ we expected the preventive ablation to reduce the risk of receiving appropriate ICD therapy by 65%. On the assumption of an equal enrolment ratio, we calculated that 58 patients are required to achieve 80% power with a two-sided error of 0.05.

Categorical variables are represented as frequencies and percentages, and were compared using χ^2^ and Fisher’s exact tests as appropriate. The normality of distribution was tested with the Kolmogorov–Smirnov test. Continuous variables are presented as mean ± standard deviation or as median and interquartile range (IQR). The outcome analysis was performed according to the intention-to-treat principle and all the data obtained between the randomization date and the end of the study were included. Intergroup differences were compared using an independent sample Student’s *t*-test or Wilcoxon rank-sum test as appropriate. Intergroup differences in the time-to-event outcomes were assessed using the log-rank test and the Cox proportional hazards model. In addition to univariate analysis, the multivariate analysis was performed with prespecified covariates of LVEF, functional capacity according to New York Heart Association classification (NYHA class), number of guideline-directed HF medications, and the presence of cardiac resynchronization therapy (CRT). A two-sided *P*-value of 0.05 was considered statistically significant. Statistical analysis was performed in IBM SPSS Statistics for Windows version 22.0 (IBM Corp., Armonk, NY, USA).

## Results

### Patient population

**Figure 1:**
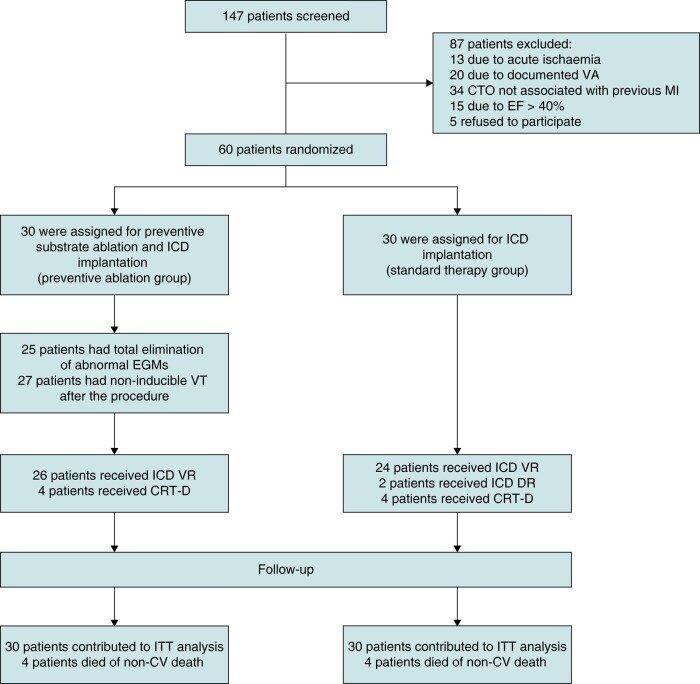
Study flowchart. Randomization and allocation of patients are shown. VA, ventricular arrhythmia; CTO, chronic total occlusion; MI, myocardial infarction; EF, ejection fraction; EGM, electrogram; VT, ventricular tachycardia; ICD VR, single-chamber implantable cardioverter-defibrillator; ICD DR, dual-chamber implantable cardioverter-defibrillator; CRT-D, cardiac resynchronization therapy with a defibrillator; ITT, intention-to-treat; CV, cardiovascular.

From September 2017 to February 2022, we enrolled 60 patients. Thirty patients were randomized to the standard therapy group with ICD implantation and 30 patients to the preventive ablation group with preventive substrate homogenization and subsequent implantation of ICD (*Figure 1*). Baseline characteristics are presented in *Table 1* and were similar in both study groups. Most enrolled patients were males (91.7%). Previous symptomatic MI was reported in 48.3% of patients and 50% of patients underwent previous myocardial revascularization. The median time interval between previously documented MI and randomization was 79 months (IQR 125). To determine non-viable myocardium, SPECT was used only in 10 patients; 6 were randomized to the preventive ablation arm and 4 to the standard therapy arm. The majority of patients (73.3%) had IRA-CTO of one coronary artery, most frequently of right coronary artery (55%), followed by left circumflex artery (28.3%). None of the patients had any attempts of additional IRA-CTO coronary artery revascularization.

**Table 1:** Baseline characteristics

Characteristic	Standard therapy group (*n* = 30)	Preventive ablation group (*n* = 30)
Age at enrolment, y (IQR)	71 (10)	65 (16)
Male sex, *n* (%)	26 (86.7)	29 (96.7)
Previous symptomatic MI, *n* (%)	16 (53.3)	13 (43.3)
Previous percutaneous revascularization, *n* (%)	13 (43.3)	13 (43.3)
Previous surgical revascularization, *n* (%)	4 (13.3)	1 (3.3)
No. of CTOs		
One vessel	21 (70%)	23 (76.7%)
Two vessels	9 (30%)	7 (23.3%)
IRA-CTO location		
RCA	20 (66.7)	13 (43.3)
LAD	6 (20)	8 (26.7)
LCX	6 (20)	11 (36.7)
LVEF, % (IQR)	34 (8)	37 (9)
New York Heart Association class, *n* (%)		
I	1 (3.3%)	1 (3.3%)
II	17 (56.7%)	20 (66.7%)
III	12 (40%)	9 (30%)
IV	0	0
Atrial fibrillation, *n* (%)	10 (33.3)	9 (30)
Hypertension, *n* (%)	25 (83.3)	24 (80)
Chronic kidney disease, *n* (%)	6 (20)	6 (20)
Diabetes mellitus, *n* (%)	9 (30)	8 (26.7)
Heart failure medication, *n* (%)		
ACE inhibitor or ARB	16 (53.3)	13 (42.3)
ARNI	12 (40)	16 (53.3)
Beta-blocker	29 (96.7)	29 (96.7)
MRA	27 (90)	21 (70)
Diuretic	14 (46.7)	18 (60)

ACE, angiotensin-converting enzyme; ARB, angiotensin receptor blocker; ARNI, angiotensin receptor-neprilysin inhibitor; CTO, chronic total occlusion; IRA-CTO, infarct-related artery-CTO; IQR, interquartile range; LAD, left anterior descending artery; LCX, left circumflex artery; MI, myocardial infarction; MRA, mineralocorticoid receptor antagonist; *n*, number; RCA, right coronary artery; y, years.

Median LVEF was 34% (IQR 8), and most patients were in NYHA Classes II and III (61.7 and 35%, respectively). All patients were receiving guideline-directed HF therapy which was well optimized (*Table 1*). None of the included patients were on amiodarone at enrolment and after the ablation procedure or ICD implantation.

### Procedures

Substrate catheter ablation was performed in all 30 patients randomized to the preventive ablation group. In two (6.7%) patients additional ablations were performed outside the area of IRA-CTO-related substrate. Total elimination of abnormal EGMs was achieved in 26 (86.7%) patients. At the end of the procedure, VA was not inducible in 27 (90%) patients. There were two (6.6%) major complications associated with the ablation procedure. One patient underwent CRT implantation due to the iatrogenic complete atrioventricular block and one patient suffered a post-procedural ischaemic stroke which resolved without consequences. There were no differences in procedural characteristics and success rates between the two centres performing catheter ablation procedures. Detailed comparison is available in the Supplementary material online.

All 60 patients enrolled in the study received an ICD device. There were no complications related to the implantation procedure. In the preventive ablation group, 26 patients received a single-chamber ICD, and 4 patients received a cardiac resynchronization ICD device (CRT-D). In the standard therapy group 26 patients received single-chamber device, 2 a dual-chamber ICD, and 4 patients a CRT-D device.

### Clinical outcomes

**Figure 2:**
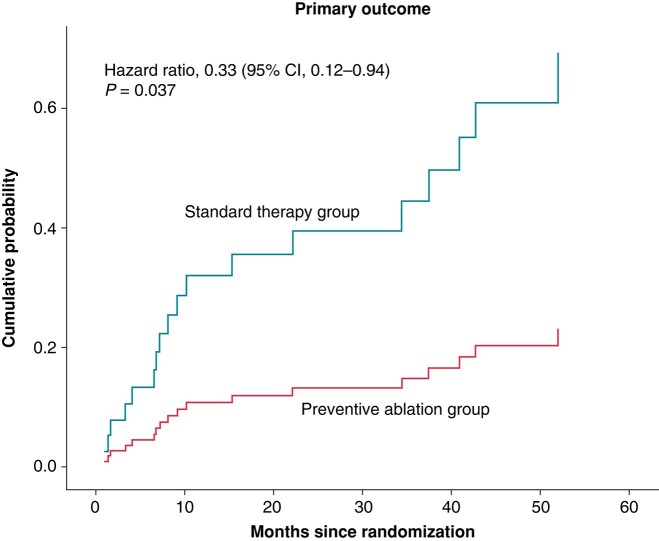
Primary outcome. Cumulative probability of the primary endpoint composed of appropriate ICD therapy or unplanned hospital admission due to ventricular arrhythmia.

The mean follow-up time was 44.7 ± 20.7 months and did not differ between groups (*P* = 0.22). The primary endpoint of appropriate ICD therapy or unplanned hospital admission due to symptomatic VAs occurred in 13 (43.3%) patients randomized to the standard therapy group and in 5 (16.7%) patients randomized to the preventive ablation group (HR 0.33, 95% CI 0.12–0.94) (*Figure 2*, *Table 2*).

**Table 2:** Primary and secondary outcomes

Outcome	Standard therapy group (*N* = 30)	Preventive ablation group (*N* = 30)	Hazard ratio (95% CI)	*P* value	
				Cox regression	Log-rank test
Primary composite outcome, *n* (%)	13 (43.3)	5 (16.7)	0.33 (0.12–0.94)	0.037	0.028
Appropriate ICD therapy, *n* (%)	12 (40)	5 (16.7)	0.37 (0.13–1.05)	0.061	0.051
Unplanned hospital admission for symptomatic VAs, *n* (%)	9 (30)	0 (0)	n/a	n/a	0.001
Unplanned cardiac hospital admission, *n* (%)	16 (53.3)	4 (13.3)	0.21 (0.07–0.63)	0.006	0.002
Electrical storm, *n* (%)	6 (20)	0 (0)	n/a	n/a	0.01
Cardiovascular death, *n* (%)	8 (26.7)	4 (13.3)	0.41 (0.12–1.38)	0.151	0.139
Heart failure hospitalization, *n* (%)	10 (33.3)	4 (13.3)	0.36 (0.11–1.16)	0.087	0.074
Death from any cause, *n* (%)	12 (40)	8 (26.7)	0.55 (0.22–1.37)	0.200	0.194

ICD, implantable cardioverter-defibrillator; *n,* number of patients; VF, ventricular fibrillation; VT, ventricular tachycardia.

**Figure 3:**
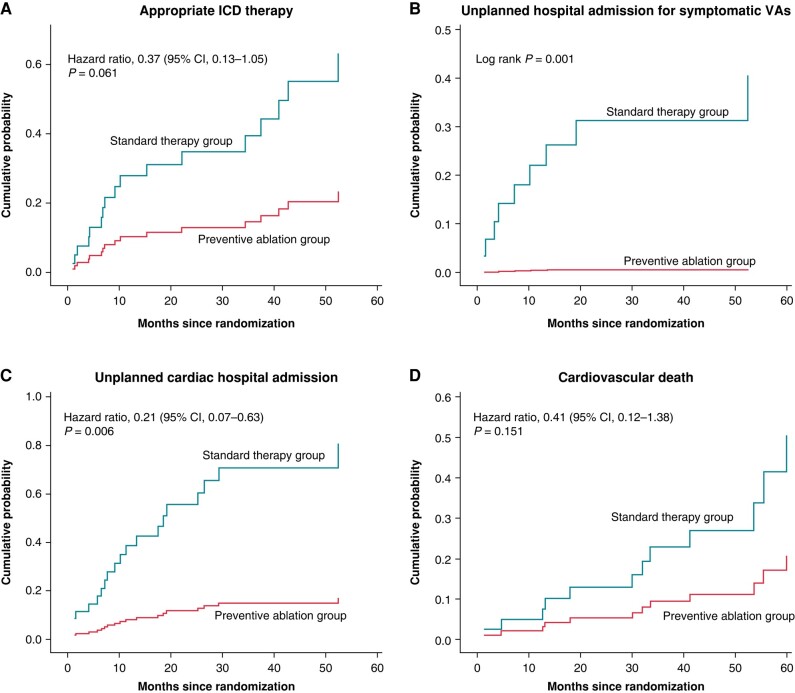
Secondary outcomes. Cumulative probabilities for the secondary outcomes of appropriate ICD therapy (*A*), unplanned hospital admission for ventricular arrhythmia (*B*), unplanned cardiac hospital admission (*C*), and cardiovascular death (*D*). ICD, implantable cardioverter-defibrillator; VA, ventricular arrhythmias.

Appropriate ICD therapy occurred in 12 (40%) patients in the standard group and 5 (16.7%) patients in the ablation group (HR 0.37, 95% CI 0.13–1.05) (*Figure 3*). While 9 patients (30%) in the standard therapy group underwent unplanned hospital admission due to VAs, there were no unplanned arrhythmia-related hospitalizations in the ablation group (*P* = 0.001). Most VA-related hospitalizations in the standard group were related to electrical storms experienced by 6 patients (20%), while there was no electrical storm after preventive substrate homogenization (*P* = 0.01). None of the electrical storm episodes was associated with acute coronary syndrome.

Unplanned cardiac hospital admission occurred in 16 patients (53.3%) in the standard group and 4 patients (13.3%) in the ablation group (HR 0.21, 95% CI, 0.07–0.63). The hazard ratios for the effect of preventive ablation on the first hospitalization for HF and CV death were 0.36 (95% CI 0.11–1.16) and 0.41 (95% CI 0.12–1.38), respectively (*Figure 3*). The total number of hospitalizations for HF was 12 in the standard group and 7 in the ablation group (*P* = 0.098). Of the 8 CV deaths in the standard group, 4 deaths were attributed to arrhythmic causes. In contrast, there were no arrhythmia-related deaths in the preventive ablation group. A total of 12 patients (40%) in the standard group and 8 patients (26.7%) in the preventive ablation group died during follow-up (HR 0.55; 95% CI, 0.22–1.37). There were no differences in primary and secondary outcomes between the two centres performing catheter ablation procedures. Detailed comparison is available in the Supplementary material online.

Multivariate regression models combining prespecified covariates of LVEF, NYHA class, number of guideline-indicated HF medications, and the presence of CRT showed similar statistical results across all the study outcomes (*Table 3*). Plots are presented in the Supplementary material online.

**Table 3:** Multivariate regression analysis of the study outcomes

Outcome	Hazard ratio (95% CI)	*P* value
Primary composite outcome	0.32 (0.11–0.91)	0.032
Appropriate ICD therapy	0.35 (0.12–1.02)	0.054
Unplanned cardiac hospital admission	0.22 (0.07–0.68)	0.009
Cardiovascular death	0.46 (0.14–1.57)	0.217
Heart failure hospitalization	0.37 (0.11–1.22)	0.103
Death from any cause	0.59 (0.24–1.49)	0.267

ICD, implantable cardioverter-defibrillator.

The total number of sustained VTs and VAs terminated by appropriate ICD therapy was higher in the standard therapy group (62 versus 10, *P* = 0.016) (*Table 4*). In total, 10 patients in the ICD group and 5 patients after substrate modification experienced appropriate ICD shock (*P* = 0.136). The number of appropriate shocks was 38 in the ICD-only group and 9 in the preventive ablation group (*P* = 0.121).

**Table 4:** Ventricular arrhythmia episodes and appropriate ICD therapies in patients receiving ICD (standard therapy group) and patients undergoing prior substrate modification (preventive ablation group)

Outcome	Standard therapy group	Preventive ablation group	*P* value
All VA episodes	62	10	0.016
VA episodes with appropriate ICD therapy	42	10	0.04
VA episodes terminated by ATP	18	5	0.453
VA episodes terminated by shock	24	5	0.088
VT episodes bellow therapy zone	20	0	0.003
All appropriate ICD therapies	75	17	0.039
ATP	37	8	0.141
Shocks	38	9	0.121

ATP, anti-tachycardia pacing; ICD, implantable cardioverter-defibrillator; VA, ventricular arrhythmia; VT, ventricular tachycardia.

During follow-up, VT ablation procedure was performed in 4 (13.3%) patients in the standard therapy group due to electrical storms. In the preventive ablation group, additional substrate ablation was performed in 2 (6.7%) patients after VA recurrence. In both cases, the epicardial approach was additionally used. Due to several recurrent VAs or electrical storms in the ICD group, amiodarone was prescribed to 9 (30%) patients and discontinued in 3 after a successful ablation procedure. In the preventive ablation group, only 1 (3.3%) patient received amiodarone.

## Discussion

This is the first randomized multicentre study showing that preventive ablation of the coronary CTO-related substrate in patients undergoing primary ICD implantation is associated with the reduced risk of appropriate ICD therapy or unplanned hospitalization due to VAs. With the acceptable incidence of major adverse events related to the ablation procedure, patients in the preventive ablation group also had fewer defibrillator interventions, arrhythmia-related hospitalizations, electrical storms, and cardiac hospitalizations.

### Study outcomes

Current guidelines give a Class IIb recommendation for catheter ablation in ICM patients who are eligible for ICD therapy after the first VA episode just before or immediately after ICD implantation.^9^ The recommendation is based on four prospective randomized trials which showed that preventive ablation in patients with a secondary prevention ICD indication for ICM and documented VA at the time of ICD implantation was associated with lower incidence of VAs and ICD interventions.^6,10–12^ Despite the fact that the letter studies did not present a clear link between improved outcomes and ablation, there is a clear body of evidence suggesting that deferred ablation after several ICD interventions is associated with a worse prognosis, lower success rates, and more periprocedural complications.^22,23^ In the study by Santangeli *et al.*^22^ VA recurrence was associated with a higher risk of death. Moreover, only 22% of deaths were attributed to arrhythmia, while the rest were associated with comorbidities, mainly HF progression.^22^ Arrhythmias in this setting may be an indication of time-dependent worsening HF rather than a change in the underlying arrhythmic substrate. Therefore, failure to show mortality benefits after ablation may simply lay in the patient selection as advanced HF is likely to result in early death regardless of arrhythmic status. This was reflected in the VTACH trial (Substrate Modification in Stable Ventricular Tachycardia in Addition to ICD therapy) where there was no benefit of substrate ablation in patients with severely impaired LV function^10^ and in a retrospective analysis of 2061 patients with less advanced underlying cardiomyopathy by Thung *et al.*^23^ where freedom from recurrent VAs after ablation was strongly associated with a significant improvement in survival. Similarly, the PARTITA trial,^13^ which was the only prospective trial specifically addressing the timing of catheter ablation relative to the first ICD shock was associated with improved clinical outcomes. Therefore, substrate homogenization early in the disease progression may be a more effective strategy to improve VA-related clinical outcomes.

In the retrospective study by Hayashi *et al.*^24^ prophylactic substrate ablation reduced the incidence of appropriate ICD therapies in primary prevention patients. Our prospective study is in line with these results and shows a significant reduction in cardiac hospitalizations due to HF or VA arrhythmias. Furthermore, we did not record any VA-related hospitalizations or electrical storms in the preventive ablation group which reflects the recent findings of the VANISH trial (Ventricular Tachycardia Ablation versus Escalated Antiarrhythmic Drug Therapy in Ischaemic Heart Disease) sub-analysis where the benefit of catheter ablation was shown in patients presenting with electrical storms.^25^

The number of HF hospitalizations, CV deaths, and deaths from any cause was numerically lower in the preventive ablation group, but statistical significance was not reached as the study was not powered to detect these secondary outcomes. Thus, further randomized studies are needed to confirm the link between preventive substrate ablation and mortality in the primary prevention setting.

### Study population

Identifying ICM patients at higher arrhythmic risk who might benefit from the preventive ablation strategy was crucial for the present study. In HF patients LVEF is a marker of disease severity but has low specificity and sensitivity to predict arrhythmic events.^26^ In contrast, ICM combined with post-MI scar which is frequently present in CTO patients presents a strong arrhythmogenic substrate.^15–17^ Di Marco *et al.*^18^ reported that 53% of primary prevention ICD recipients with IRA-CTO experienced VAs (predominantly fast VTs) during the median follow-up of 33 months. The incidence of VA occurrence was in line with our results as the primary endpoint of appropriate ICD therapy or unplanned hospitalization due to VA was registered in 13 patients (43.3%), confirming that the selected study population is indeed at high risk of VA occurrence. Furthermore, despite the conservative device programming with primary prevention settings in our study population, more than half of all recorded VAs were treated with ICD shocks.

### Substrate ablation procedure

Substrate homogenization with complete abolition of abnormal EGMs effectively reduces VA recurrence.^7,13,27^ In our study, we were able to achieve complete elimination of all abnormal potentials in 83% of the patients, which is comparable to the studies that were exploring preventive ablation strategy (86 and 100%)^7,13^ and higher than in the retrospective study which included patients presenting with multiple VAs where the success rate of substrate modification was only 52%.^28^ As the data come from the experienced centre, we can hypothesize that patients in whom complete scar modification is achieved may have a more accessible and less extensive substrate, reflecting a less severe disease. Contrary to our results, in a similar patient population with IRA-CTO, the observational study by Di Marco *et al.*^29^ reported a high VA recurrence despite substrate ablation. This discrepancy with our results, where the clear reduction in the primary endpoint and the incidence of VAs were observed, may be explained by the variations in the substrate ablation strategy that did not include total abolition of abnormal EGMs (including high-density remapping) as a procedural endpoint. This explanation can be extrapolated to several multicentre studies exploring preventive catheter ablation like VTACH trial^10^ and Substrate Modification Study trial,^11^ where ablation protocols were heterogeneous, allowing variations among centres, and without clear substrate homogenization endpoint.

### Safety profile

Therapies that are considered in primary prevention patients need to be safe. In our study, we recorded two (6.6%) major adverse events related to the ablation procedure. This safety profile is reassuring and in line with previous randomized trials evaluating early substrate-based ablation strategy where complication rates ranged between 2.8 and 8%^6–8,10–14^ and recent prospective multicentre registry.^27^ Several factors may contribute to the lower complication risk in case catheter ablation is considered as a primary prevention strategy. First, the substrate ablation procedure has a predictable workflow as all primary prevention patients are subjected to preprocedural imaging. Second, contrary to clinical VT ablation, substrate homogenization is typically performed in stable conditions and does not require multiple tachycardia inductions and mapping that could be poorly tolerated, especially in patients with advanced ICM.^22^ Third, scar homogenization has no negative impact on post-procedure LV function as shown in the VISTA (Ablation of Clinical Ventricular Tachycardia vs Addition of Substrate Ablation on the Long-term Success Rate of VT Ablation) study.^7^

### Clinical implications

In everyday clinical practice, the VT ablation procedure is still regarded as a last resort in the management of patients with ICM. However, ablation at the time of ICD implantation or after first appropriate ICD therapy in patients with a lower burden of comorbidities and less advanced cardiomyopathy may be associated with fewer periprocedural complications and improved clinical outcomes.^22–24,27^ Our study shows that substrate homogenization can be an effective and safe treatment option in primary prevention patients. In addition, our study also highlights the importance of identifying ICM patients with a high risk of developing VAs in whom ablation procedures might prevent the occurrence of VA and consequent debilitating ICD shocks while outweighing the potential procedural complication risk. In the future, studies that explore not only more efficient and safer catheter ablation technologies, but also newer imaging modalities, utilizing artificial intelligence, and employing interdisciplinary approaches, are necessary to potentially establish VT ablation procedures as a primary prevention strategy.

### Limitations

We acknowledge some limitations of the study. First, despite being a multicentre trial, only two high volume centres were performing VT ablations, which may limit the generalization of our findings. Second, our study included a predominantly male population with IRA-CTO and the results cannot be extrapolated to other primary prevention patients with structural heart disease. Third, only the endocardial approach for ventricular high-density mapping and ablation was undertaken in our study. Although the epicardial approach could have produced even higher efficacy of the ablation procedure,^30^ it could have also increased the probability of additional procedure-related adverse events.^31^ In addition, according to a recent survey of European electrophysiology centres, 53% of the centres use epicardial access only after the failed previous endocardial ablation,^32^ which reflects the approach of the present study. Fourth, MRI was not readily available in some participating centres during the initial period of recruitment. Thus, SPECT which offers lower spatial resolution compared to MRI was used to determine non-viable myocardium. However, in all six included patients, randomized to the preventive ablation arm CTO-related substrate was confirmed during LV mapping, while among four patients in the standard arm, one patient had catheter ablation due to electrical storm. Therefore, it is conceivable that the imaging modality used to determine CTO-related substrate did not have a meaningful impact on the study outcomes. Lastly, differences in device programming between different manufacturers may have affected the number of appropriate ICD therapies. However, the programming followed general recommendations^21^ and, thus, reflected everyday clinical practice.

## Conclusions

Preventive ablation of the coronary CTO-related substrate in patients undergoing primary ICD implantation is associated with a reduced risk of appropriate ICD therapy or unplanned hospitalization due to VAs. Preventive substrate homogenization was associated with fewer defibrillator interventions, arrhythmia-related hospitalizations, and electrical storms; however, the study was not powered to detect the reduction of cardiovascular and total mortality. Further adequately powered clinical trials are therefore needed to address the impact of preventive catheter ablation on the mortality endpoints.

## Supplementary Material

euae109_Supplementary_Data

## Data Availability

The data underlying this article are available in the article and in its online supplementary material.
