# Supplementary material for: Impact of preventive substrate catheter ablation on implantable cardioverter-defibrillator interventions in patients with ischaemic cardiomyopathy and infarct-related coronary chronic total occlusion: The PREVENTIVE VT randomized multicentre trial
Source: Europace. 2024 Apr 24;26(5):euae109. doi: 10.1093/europace/euae109 (PMC11086562; doi:10.1093/europace/euae109)
Supplement: euae109_Supplementary_Data [file euae109_supplementary_data.docx]

**Supplementary data**

**Participating centers**

1. *Department of Cardiology – University Medical Centre Ljubljana* (coordinating center): high volume PCI/CTO interventions, high volume device implantations, high volume VT ablations (>50 per year).
2. *Cardiovascular surgery department – University Medical Centre Ljubljana*: not performing PCI/CTO, high volume device implantations, high volume VT ablations (>50 per year).
3. *General hospital Celje*: experienced in PCI/CTO interventions, high volume device implantations, not performing VT ablations.
4. *General hospital Izola*: high volume PCI/CTO interventions, high volume device implantations, not performing VT ablations.

If a study participant was assigned to preventive substrate ablation arm after randomization in a center which did not perform the VT ablation procedure, the patient was transferred to either the Department of Cardiology at UMC Ljubljana or the Department of Cardiovascular Surgery department at UMC Ljubljana for the ablation procedure. After the ablation, the patient returned to the referring center for ICD device implantation. Further device follow-ups were then performed at the referring center.

**Detailed inclusion and exclusion criteria**

**Inclusion criteria:**

1. Ischemic cardiomyopathy with reduced ejection fraction (EF ≤ 40%) estimated by cardiac MRI or echocardiography within 30 days before enrollment
2. Coronary Chronic Total Occlusion (CTO) associated with a previous MI confirmed by coronary angiography and late gadolinium enhancement MRI or myocardial perfusion imaging within 30 days before enrollment
3. Implantable cardioverter-defibrillator (ICD) indication for primary prevention
4. Patient has provided written informed consent

**Exclusion criteria:**

1. Age < 18 years or > 85 years
2. Documented sustained ventricular tachycardia before enrollment
3. Class IV New York Heart Association (NYHA) heart failure
4. CTOs not associated with a prior infarction in their territory
5. Acute myocardial infarction (MI) or acute coronary syndrome
6. Subjects with active ischemia that are eligible for revascularization
7. Documented history of MI less than 6 months before enrollment
8. Patients requiring chronic renal dialysis
9. Thrombocytopenia or coagulopathy
10. Pre-existing implantable cardioverter-defibrillator (ICD)
11. Pregnancy or breastfeeding women
12. Acute illness or active systemic infection
13. Life expectancy less than 12 months
14. Unwillingness to participate or lack of availability for follow-up
15. Valvular heart disease or mechanical heart valve precluding access to the left ventricle

**Catheter ablation procedure**

Myocardial scar (CTO-IRA) and border zone were delineated by voltage mapping in sinus rhythm utilizing 3-dimensional (3D) electroanatomic mapping system (CARTO^®^ 3, Biosense Webster, Irvine, CA, USA). A high-density voltage map of the LV was performed with a multipolar mapping catheter (PENTARAY^®^, Biosense Webster, Irvine, CA, USA). Minimum number of 500 acquisition points within the area of interest were required.

Protocol:

1. Normal myocardium, dense scar, and border zone characterization by bipolar voltage criteria as follows: <0.5 mV identifies dense scar, bipolar voltage of 0.5 to 1.5 mV border zone, and bipolar voltage >1.5 mV normal myocardium (1).
2. All local abnormal ventricular electrograms within the scar and the border zone cataloged and tagged on a 3D electroanatomical map of the LV. These abnormal local ventricular electrograms could represent myocardial channels responsible for re-entrant VTs and are the main target for ablations in this study. Local abnormal ventricular activities (LAVA) share characteristic bipolar electrograms. Multiple-component low-voltage (amplitude of <0.5 mV) abnormal electrograms without an isoelectric segment and duration >130 ms were characterized as fractionated electrograms. Potentials occurring after the QRS complex and separated from the ventricular electrogram by an isoelectric interval of >20 ms were defined as late isolated potentials (2-4). In addition, abnormal decrement-evoked local ventricular electrograms unmasked during pacing with ventricular extrastimuli are also targeted for ablation (5).
3. Catheter ablation should be performed at all sites of abnormal EGMs (if safe / reachable) with the aim to eliminate all abnormal activities within the scar and scar border. Catheter ablation is performed by means of radiofrequency (RF) current, with the irrigated-tip, contact force sensing ablation catheter (SMARTTOUCH^®^ and SMARTTOUCH^®^ SF, Biosense Webster, Irvine, CA, USA). No time limit is imposed for mapping and RF ablation, which remained at operator’s discretion.
4. At the end of ablation procedure, high-density LV remapping is requred for potential residual abnormal EGMs.
5. Finaly, programmed stimulation from the RV apex with up to 3 extrastimuli need to be performed to test potential VA inducibility.

Endpoints of the ablation procedure were:

- complete elimination of local abnormal ventricular electrograms within the scar and scar border after high-density re-mapping
- non-inducibility of monomorphic VT after programmed stimulation from the RV apex with up to 3 extrastimuli delivered

References:

1. Marchlinski FE, Callans DJ, Gottlieb CD, Zado E. Linear ablation lesions for control of unmappable ventricular tachycardia in patients with ischemic and nonischemic cardiomyopathy. Circulation 2000; 101:1288–96.
2. Cassidy DM, Vassallo JA, Buxton AE, Doherty JU, Marchlinski FE, Josephson ME. The value of catheter mapping during sinus rhythm to localize site of origin of ventricular tachycardia. Circulation 1984; 69:1103–10.
3. Cassidy DM, Vassallo JA, Miller JM, et al. Endocardial catheter mapping in patients in sinus rhythm: relationship to underlying heart disease and ventricular arrhythmias. Circulation 1986; 73:645–52.
4. Harada T,Stevenson WG, Kocovic DZ, et al. Catheter ablation of ventricular tachycardia aftermyocardial infarction: relation to endocardial sinusrhythm late potentials to the reentry circuit. J Am Coll Cardiol 1997; 30:1015–23.
5. Jais P, Maury P, Khairy P, et al. Elimination of local abnormal ventricular activities: a new end point for substrate modification in patients with scar-related ventricular tachycardia. Circulation 2012; 125:2184–96.

**Definitions of complications related to catheter ablation procedure**

Major complications were defined as those that could be attributed to the ablation procedure (usually during the same hospital stay) and requiring intervention or prolonging hospitalization. These include: periprocedural death or cardiac arrest, vascular access complications requiring percutaneous or surgical intervention, oesophageal fistula, stroke, or transient ischaemic attack (TIA), systemic embolism, pericardial perforation, pericardial effusion or tamponade requiring percutaneous or surgical intervention, myocardial infarction, and permanent heart block requiring pacing device implantation.

**Procedural characteristics and success rates according to the ablation center**

| **Variable** | **Center I**  **(N=18)** | **Center II**  **N=12** | **P value** |
| --- | --- | --- | --- |
| **Points mapped - n±SD** | 652±137 | 624±102 | 0.552 |
| **Ablation points - n±SD** | 109±40 | 125±46 | 0.343 |
| **Procedural time - min±SD** | 307±74 | 349±112 | 0.228 |
| **Total elimination of abnormal EGMs - n (%)** | 16 (88.9%) | 10 (83.3%) | 1.0 |
| **VT non-induciblity - n (%)** | 17 (94.4%) | 10 (83.3%) | 0.548 |

*Legend: n - number, EGM - electrogram, SD - standard deviation, VT - ventricular tachycardia.*

**Primary and secondary outcomes according to the ablation center**

| **Outcome** | **Center I (N=18)** | **Center II (N=12)** | **P Value** |
| --- | --- | --- | --- |
|  |  |  |  |
| **Primary composite outcome - n (%)** | 4 (22.2%) | 1 (8.3%) | 0.391 |
| Appropriate ICD therapy - n (%) | 4 (22.2%) | 1 (8,3%) | 0.391 |
| Unplanned hospital admission for symptomatic VAs - n (%) | 0 | 0 |  |
| **Unplanned cardiac hospital admission - n (%)** | 3 (16.7%) | 1 (8.3%) | 0.580 |
| **Electrical storm - n (%)** | 0 | 0 |  |
| **Cardiovascular death - n (%)** | 3 (16.7%) | 1 (8.3%) | 0.667 |
| **HF hospitalization - n (%)** | 3 (16.7%) | 1 (8.3%) | 0.580 |
| **Death from any cause - n (%)** | 4 (22.2%) | 4 (33.3%) | 0.442 |
| **Major complication - n (%)** | 1 (5.6%) | 1 (8.3%) | 1 |

*Legend: ICD - implantable cardioverter-defibrillator, n - number of patients, VF - ventricular fibrillation, VT - ventricular tachycardia, HF - heart failure.*

**Device programming - primary prevention ICD settings for different manufacturers**

Most patients received single chamber (SC) ICD devices. The manufacturer and other types of devices were at the physician's discretion. Dual chamber (DC) devices were programmed to allow intrinsic rhythm and resynchronization devices with defibrillator (CRT-Ds) were programmed on DDD (R) 45 – 130 / 140 bpm. Uniform primary prevention device settings for arrhythmia detection and therapy from different manufacturers was advised according to the recommendations (Table) from Wilkoff BL, et al. 2015 HRS/EHRA/APHRS/SOLAECE expert consensus statement on optimal implantable cardioverter-defibrillator programming and testing. Heart Rhythm. 2016 Feb;13(2):e50-86.

In case of recorded VT below the detection zone during follow-up, VT zone was programmed 10 – 20 bpm below the rate of recorded VT.

**Table representing the recommended programming for ICD devices.**

| **Company** | **Brady**  **SC ICD** | **VT** | **VT 2** | **VF** | **Therapy** | **Discriminators** |
| --- | --- | --- | --- | --- | --- | --- |
| **Medtronic** | **VVI 40 bpm** | **Monitor** | **OFF** | **188 bpm**  **30/40 beats** | **VF: ATP before charging; shocks full output** | **SC: morphology (Wavelet)**  **DC / CRT-D: PR Logic, Wavelet** |
| **St. Jude Medical / Abbott** | **VVI 40 bpm** | **Monitor** | **187 bpm**  **30 intervals** | **250 bpm**  **30 intervals** | **VF: ATP while charging, shocks**  **VT2: ATP, ≥1 burst of 8 pulses 85% VT CL, shocks on** | **SC: morphology**  **DC / CRT-D: Far-Field Morphology, Arrhythmia onset, Interval Stability** |
| **Biotronik** | **VVI 40 bpm** | **Monitor** | **188 bpm**  **30 intervals** | **231 bpm**  **30 / 40 intervals** | **VF: ATP one-shot, 1 burst of 8 pulses at 88% CL, full output shocks**  **VT2: ≥ 1ATP bursts of 8 pulses at 88%CL, shocks on** | **SC: morphology (MorphMatch)**  **DC / CRT-D: SMART** |
| **Boston Sci** | **VVI 40 bpm** | **Monitor** | **185 bpm**  **8/10 intervals + 12 sec duration** | **250 bpm**  **8/10 intervals + 5 sec duration** | **VF: ATP in VF zone, shocks max output**  **VT: ATP-1: Scan, ≥1 bursts, 8 pulses at 84% CL, ATP-2: OFF, shocks on** | **SC: morphology (RhythmID)**  **DC / CRT-D: morphology (RhythmID)** |

*Legends: VF – ventricular fibrillation, ATP – antitachycardia pacing, VT – ventricular tachycardia, CL – cycle length, SC – single chamber, DC – dual chamber, CRT-D – cardiac resynchronization device with defibrillator.*

**Multivariate regression model plots of study the study outcomes**

**
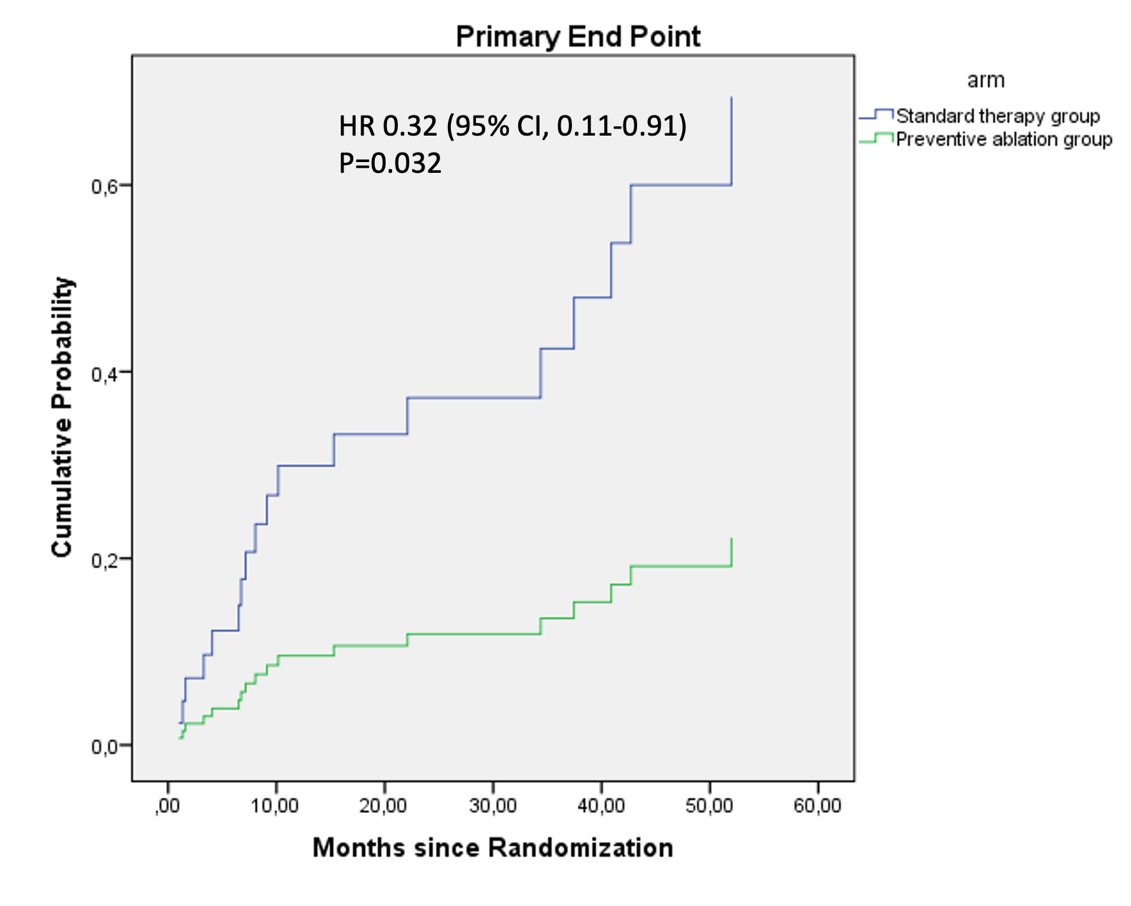
**

**
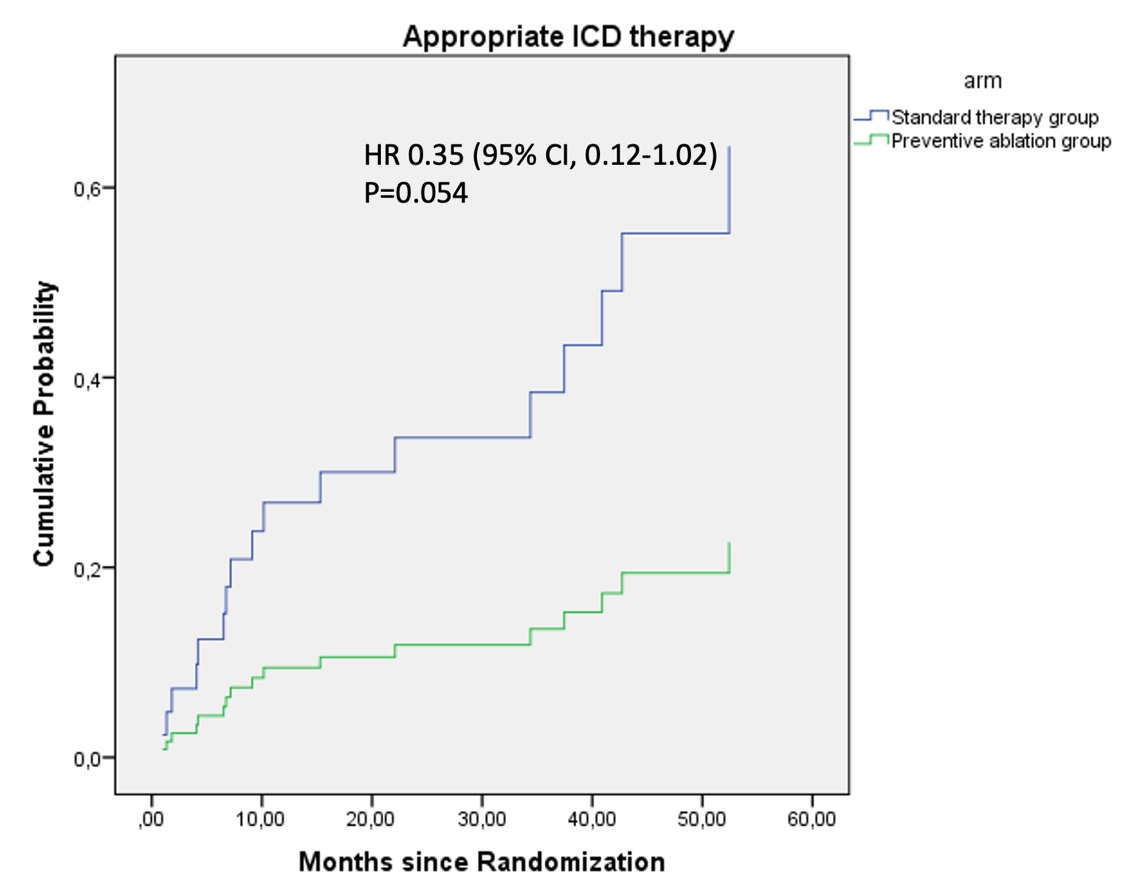
**

**
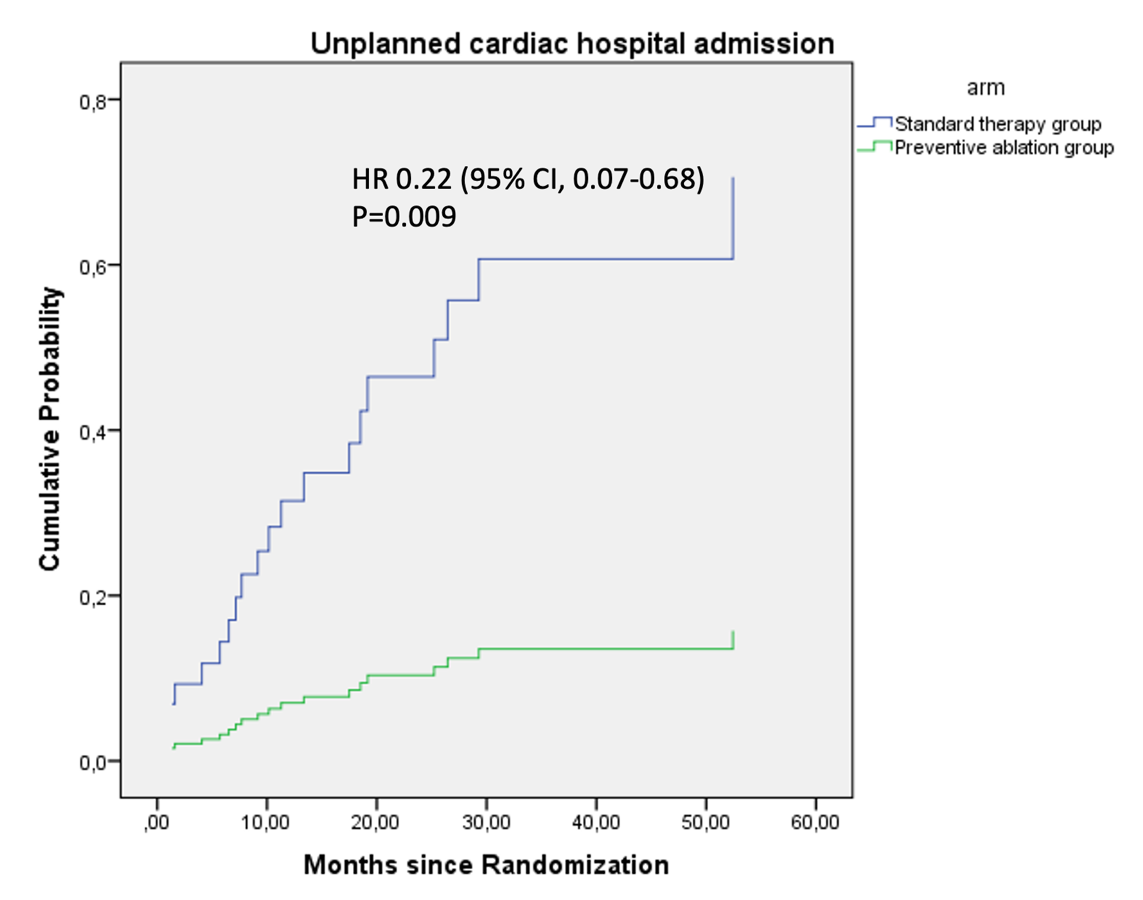
**

**
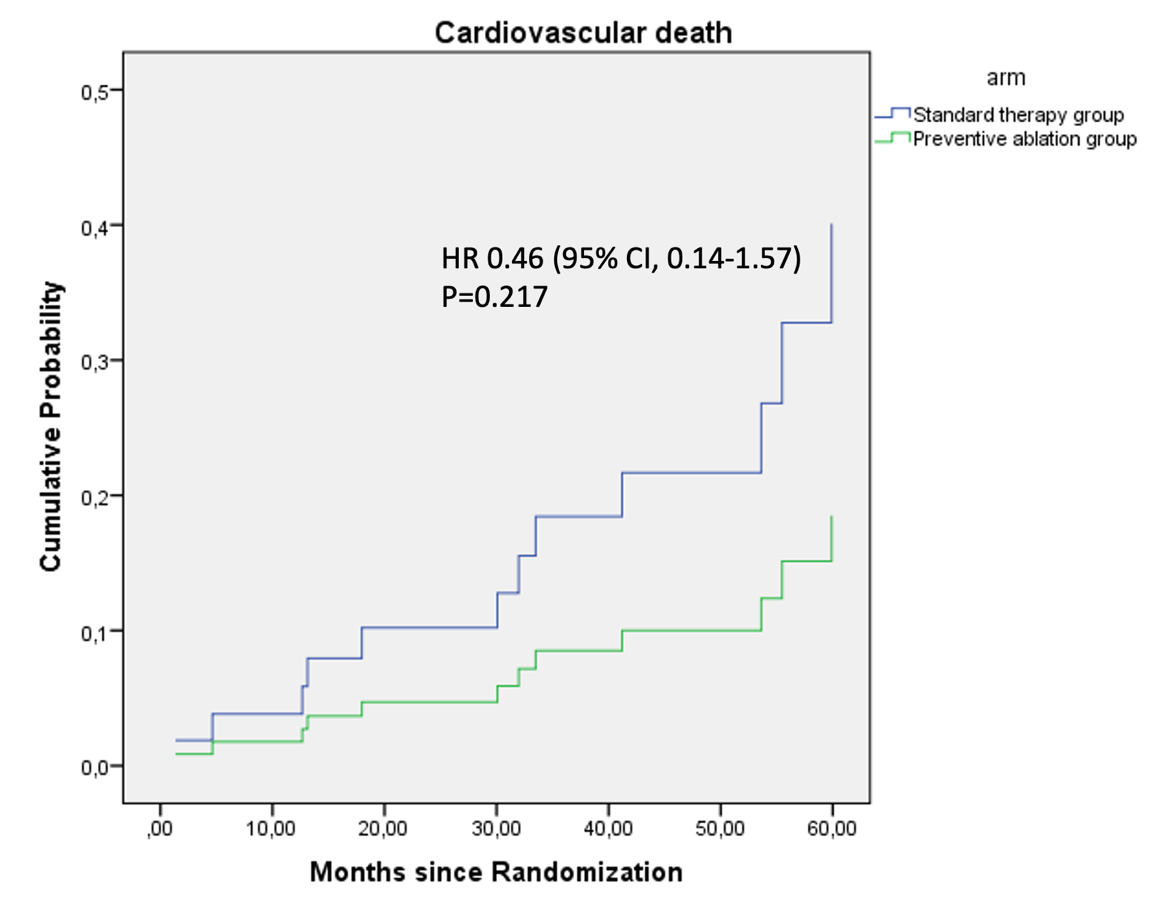
**
